# Proteomics Pipeline for Biomarker Discovery of Laser Capture Microdissected Breast Cancer Tissue

**DOI:** 10.1007/s10911-012-9252-6

**Published:** 2012-05-30

**Authors:** Ning Qing Liu, René B. H. Braakman, Christoph Stingl, Theo M. Luider, John W. M. Martens, John A. Foekens, Arzu Umar

**Affiliations:** 1 Department of Medical Oncology and Daniel Den Hoed Cancer Center, Erasmus University Medical Center, Dr. Molewaterplein 50, Be-401, P.O. Box 2040, 3000 CA Rotterdam, the Netherlands; 2 Netherlands Proteomics Center, Rotterdam, the Netherlands; 3 Center for Translational Molecular Medicine, Rotterdam, the Netherlands; 4 Department of Neurology, Erasmus University Medical Center, Rotterdam, the Netherlands; 5 Cancer Genomics Centre, Rotterdam, the Netherlands

**Keywords:** Breast cancer, High resolution mass spectrometry, Label-free proteomics, Data analysis, Estrogen receptor associated proteins

## Abstract

Mass spectrometry (MS)-based label-free proteomics offers an unbiased approach to screen biomarkers related to disease progression and therapy-resistance of breast cancer on the global scale. However, multi-step sample preparation can introduce large variation in generated data, while inappropriate statistical methods will lead to false positive hits. All these issues have hampered the identification of reliable protein markers. A workflow, which integrates reproducible and robust sample preparation and data handling methods, is highly desirable in clinical proteomics investigations. Here we describe a label-free tissue proteomics pipeline, which encompasses laser capture microdissection (LCM) followed by nanoscale liquid chromatography and high resolution MS. This pipeline routinely identifies on average ∼10,000 peptides corresponding to ∼1,800 proteins from sub-microgram amounts of protein extracted from ∼4,000 LCM breast cancer epithelial cells. Highly reproducible abundance data were generated from different technical and biological replicates. As a proof-of-principle, comparative proteome analysis was performed on estrogen receptor α positive or negative (ER+/−) samples, and commonly known differentially expressed proteins related to ER expression in breast cancer were identified. Therefore, we show that our tissue proteomics pipeline is robust and applicable for the identification of breast cancer specific protein markers.

**Electronic supplementary material:**

The online version of this article (doi:10.1007/s10911-012-9252-6) contains supplementary material, which is available to authorized users.

## Introduction

With the rapid development of high resolution mass spectrometry (MS), global screening of protein markers becomes feasible and is starting to play an important role in biomarker discovery [1]. Protein markers are more related to disease phenotype and are more targetable for therapy in comparison with transcriptome-based biomarkers. Hence, identification of sensitive and specific protein makers is of importance for clinical practice. However, to develop a reproducible workflow for the robust identification of such biomarkers, several important technical aspects have to be taken into account.

A challenge in reliable protein marker identification is the heterogeneity of tumor tissues. Tumor cells are almost always surrounded by stromal compartments and infiltrating cells and the percentage of epithelial tumor cells can vary dramatically between individual tumor samples. Laser capture microdissection (LCM) is a widely applied technique to isolate tumor cells from their surrounding tissues [2, 3], which allows enrichment of cells of interest and removes bias introduced by comparison of tumor samples with different morphology. Nevertheless, LCM is a laborious and time-consuming procedure, which means that only limited number of cells can be collected from individual samples, and is therefore difficult to apply on large cohort of tumor tissues when a large number of tumor cells per sample are needed for a successful measurement. Furthermore, a highly sensitive proteomics platform is required to analyze proteome of LCM materials in depth. Nanoscale liquid chromatography coupled to tandem mass spectrometry (nLC-MS/MS) enables identification of >1,000 proteins from sub-microgram breast cancer tissue in a 3 h gradient, and makes it possible to apply LCM for large scale biomarker discovery [4].

Secondly, the human proteome exhibits a very large dynamic range in protein expression, while MS based analysis can cover at best up to 4–5 orders of magnitude. This directly leads to reduced reproducibility for measurements of lower abundant proteins, because their corresponding peptides will not be consistently observed through all measured MS datasets, even though these peptides are biologically present through all the experimental samples, which leads to a large percentage of missing data in MS based proteomics. Moreover, sample handling steps are often complicated and need to be carefully controlled [5]. Furthermore, label-free quantification (LFQ) is often used for global screening of biomarkers but the quantitative capacity of LFQ remains a question. All these obstacles make it difficult to identify clinically valuable protein markers using an MS based proteomics approach. Great efforts have been made to improve protocols for sample preparation [6–8], to create sensitive and confident methods for multiple peak alignment, protein quantification and database searching [9, 10], and to perform more robust and reliable statistical analyses [11–14], in order to reliably identify biomarkers. Taken together, a well-designed pipeline for proteomics based biomarker discovery can greatly improve reproducibility of sample preparation, produce more quantitative data, and therefore increase the possibility of identifying reliable and clinically relevant biomarkers.

Here we describe a robust label-free tissue proteomics pipeline that is applicable for breast cancer biomarker discovery. This pipeline identified on average ∼10,000 peptides corresponding to ∼1,800 proteins from as little as ∼4,000 LCM breast cancer epithelial cells (corresponding to sub-microgram protein lysates). Obtained data were highly reproducible and quantitative, and allowed identification of more than 100 differentially expressed proteins between estrogen receptor α positive (ER+) and negative (ER−) breast tumor samples.

## Methods

### Tumor Tissues and Samples

Ten fresh frozen tumor tissues were selected from our liquid N2 bank, of which 5 were ER positive (ER+) and 5 were ER negative (ER−). ER and progesterone receptor (PR) status of 10 samples were determined by enzyme-linked immunosorbent assay. From one ER tumor sample, five laser capture microdissected control samples (LCM-CTRLs, biological replicates) were prepared using a previously described protocol [5, 7]. From the same biological source, whole tissue lysate (WTL) was prepared and measured by nLC-MS/MS for 12 times (whole tissue lysate control samples, WTL-CTRLs, technical replicates). The remaining five ER+ and four ER− samples were used as experimental samples to identify differentially expressed proteins. Detailed sampling plan and experimental design is explained in Supplementary Fig. 1. This study was approved by the Medical Ethics Committee of the Erasmus Medical Center Rotterdam, The Netherlands (MEC 02.953) and was performed in accordance to the Code of Conduct of the Federation of Medical Scientific Societies in the Netherlands.

### Isolation of Breast Tumor Epithelial Cells

Cryosectioning and LCM were performed according to previously described protocol [7]:

1. Sterilize and hydrate polyethylene naphthalate (PEN) coated glass slide (Carl Zeiss MicroImaging, GmbH, Munich, Germany) under ultraviolet light for 30 min;
2. Cut 4 to 6 8-μm tissue cryosections and attach those sections on a PEN slide;
3. Fix tissue sections in ice-cold 70 % ethanol, briefly dry slides for 30 s at room temperature, and then dehydrate in ice-cold 100 % ethanol;
4. Dry PEN slide in room temperature and place it in a plastic slide container wrapped with aluminum foil or Parafilm and store the slide container at −80 °C;
5. Prior to LCM, defrost the PEN slide at room temperate for 5 min inside a sealed slide container;
6. Rinse the PEN slide in tap water for 15 s, stain tissue sections in hematoxylin for 30 s, blue staining in tap water for 15 s, and finally dehydrate tissue sections in 50, 70, 95 and 100 % ethanol for 15 s each and 60 s for the final 100 % ethanol wash. A volume of 200 μl Halt protease and phosphatase inhibitor cocktail (100 × concentration, Thermo Fischer Scientific Inc., Rockford, IL, USA) is added into 20 ml of tap water, 50 and 70 % ethanol, respectively;
7. Isolate tumor cells using a P.A.L.M. MicroBeam system, and collect ∼500,000 μm^2^ (equivalent to ∼4,000 tumor cells [8]) in ZEISS opaque adhesive caps (Carl Zeiss MicroImaging, GmbH, Munich, Germany);
8. Suspend captured cells in 20 μl of 0.1 % RapiGest surfactant (Waters Corp., Milford, MA, USA) in 50 mM ammonium bicarbonate (SIGMA, Saint Louis, MO, USA) buffer, store sample at −80 °C.

Tip: (1) An optimal range of tumor area per dissection is between 5,000 and 25,000 μm^2^ to ensure successful catapulting; (2) Always check the entire tumor sections under the microscope at 5× magnification to ensure all LCM tumor pieces are collected in the adhesive cap; (3) After transferring captured tumor cells into a LoBind Eppendorf tube (Eppendorf, Hamburg, Germany), check the cap under microscope to ensure all LCM tumor cells are transferred into the LoBind Eppendorf tube.

### In-Solution Tryptic Digestion

In-solution tryptic digestion was performed according to the instructions of the manufacturer and as previously described [5]:

1. Lyse cell suspension in 0.1 % RapiGest buffer using an Ultrasonics Disruptor Sonifier II (Model W-250/W-450, Branson Ultrasonics, Danbury, CT) at 70 % amplitude for 1 min;
2. Denature proteins at 95 °C for 5 min;
3. Reduce denatured proteins at 60 °C for 30 min by adding dithiothreitol (SIGMA, Saint Louis, MO, USA) to a final concentration of 5 mM;
4. Alkylate reduced proteins in the dark for 30 min by adding iodoacetamide (Thermo Scientific, Rockford, IL, USA) to a final concentration of 15 mM;
5. Digest unfolded proteins at 37 °C for 4 h using MS-grade porcine modified trypsin gold (Promega, Madison, WI, USA) at a 1:4 (enzyme/protein) ratio (∼400 ng of protein can be extracted from ∼4,000 microdissected cells [1], therefore 100 ng of trypsin was used for digestion);
6. Acidify tryptic digests with 0.5 % trifluoroacetic acid (SIGMA, Saint Louis, MO, USA) and incubate mixture at 37 °C for 30 min to terminate tryptic digestion and degrade RapiGest;
7. Centrifuge digests at 14,000 rpm for 15 min at 4 °C to precipitate undissolved cellular debris and the insoluble fraction of hydrolyzed RapiGest;
8. Transfer supernatant into high performance liquid chromatography vials for nLC-MS/MS measurements.

### nLC-MS/MS Analysis

Proteomic profiling was performed on an Ultimate 3000 nLC system (Dionex, Amsterdam, The Netherlands) connected online with a hybrid linear ion trap/Orbitrap mass spectrometer (LTQ-Orbitrap-XL, ThermoElectron, Bremen, Germany) following a slightly modified procedure as described previously [8].

1. Method of sample loading: A volume of 20 μl (equivalent to ∼4,000 cells or ∼400 ng) from each sample was loaded on a trap column (PepMap C18, 300 μm I.D. × 5 mm, 5 μm particle size, 100 Å pore size; Dionex, Amsterdam, The Netherlands) for concentration and desalting using 0.1 % trifluoroacetic acid (in water) as loading solvent at a flow rate of 20 μl/min;
2. nLC systems and elution gradient: After sample loading, the trap column was switched online to directly connect with a reversed-phase 75-μm I.D. × 50-cm fused silica capillary column packed with 3-μm C18 particles (PepMap, Dionex, Amsterdam, The Netherlands). Peptides were gradually eluted out at a flow rate of 250 nl/min at 40 °C column temperature using the following binary gradient: the gradient started with 100 % mobile phase A (97.9 % H_2_O, 2 % acetonitrile, 0.1 % formic acid) to 25 % mobile phase B (80 % acetonitrile, 19.02 % H_2_O, 0.08 % formic acid) over the first 120 min, and then a steeper gradient was used to further increase mobile phase B to 50 % in the next 60 min;
3. MS systems and settings: The eluted peptides were directly sprayed with a voltage of 1.6 kV into the on-line coupled LTQ-Orbitrap-XL MS using nano electro-spray ionization source equipped with a metal-coated nano-scale emitter (New Objective, Woburn, MA). Mass spectra were acquired over a mass-to-charge ratio (m/z) range 400–1,800 Th at a resolving power of 30,000 at 400 m/z. Target of automatic gain control (AGC) was set at 10^6^ ions and lock mass set to 445.120025 m/z (protonated (Si(CH_3_)_2_O))_6_) [15]. On the basis of this full scan, the top 5 intensive ions were consecutively isolated (AGC target set to 10^4^ ions) and fragmented by collisionally activated dissociation applying 35 % normalized collision energy in the linear ion trap. Parent ions within a mass window of ±5 ppm were then excluded for MS/MS fragmentation for the next 3 min or until the precursor intensity fell below a signal-to-noise ratio of 1.5 for more than 10 scans (early expiration). Orbitrap full scan spectra and ion trap MS/MS fragmentation spectra were acquired partially simultaneously (preview mode for FTMS master scan enabled).

### Database Searching

The recorded MS spectra were analyzed by MaxQuant Software (version 1.1.1.36) [9].

1. Database searching and identification: The initial search was limited to a mass window of 7 ppm and a fragment mass window of 0.5 Th. To construct an MS/MS peak list file, up to top 8 peaks per 100 Da window were extracted and submitted to search against a concatenated forward and reverse version of the UniProtKB/Swiss-Prot human database (generated from version 2011_03, human taxonomy, 20,287 entries). Carbamidomethylation of cysteines was defined as fixed modification, while protein N-terminal acetylation and methionine oxidation were defined as variable modifications for database searching. Also, an option of second identifications was selected to allow identification of co-eluting peptides with second highest searching score from a subset of MS/MS spectra. The cutoff of false discovery rate (FDR) for peptide and protein identification was set to 0.01, and only peptides with ≥7 amino acid residues were allowed for identification. In addition, at least one unique peptide was required to identify a protein;
2. Protein quantification and multiple peak alignment: LFQ was performed by MaxQuant software on identified razor and unique peptides in order to properly quantify identified proteins. Razor peptides are non-unique peptides assigned to the protein group identified by most other peptides [16], which follows “occam’s razor” principle. Detailed methodology of LFQ algorithm was previously described in [17]. The “match between the runs” option was chosen to match the same accurate masses between multiple nLC-MS/MS runs within a retention time window of 2 min.

### Data Processing and Statistical Analysis

Data processing after MaxQuant data analysis was divided into two parts and performed as follows. A flow chart summarizes the complete strategy of statistical analyses used in our data handling pipeline (Supplementary Fig. 2).

1. Log_2_ transformation, normalization and filtering of the data:
  1.1. Peptide abundances given in the “peptides.txt” file generated by MaxQuant were first Log_2_ transformed and then median peptide abundances in individual samples were centered;
  1.2. Protein abundances normalized by LFQ algorithm integrated in MaxQuant were Log_2_ transformed for further analyses. Label free algorithm takes the maximum number of identified peptides between any two samples and compares the intensity of these peptides to determine peptide ratios. Protein abundance is computed using median values of all peptide ratios of certain protein [17];
  1.3. Peptides reserved for further analysis adhered to following criteria: (a) peptides were unique to one protein group, (b) sequences were not recognized as reversed sequences of all peptides in the database, and (c) peptides with large percentage of missing data were excluded from mixed-effect analysis of variance (ME-ANOVA) analysis. Due to the small sample cohort in this study, only peptides with abundance data in at least 5 (50 %) observations out of 9 samples were included in the ME-ANOVA analysis. In case of larger sample cohorts (e.g. *n* > 60), the threshold for peptide inclusion could be set to a minimum of 30 % observations;
2. Statistical analysis:
  In this part of data handling, we took two separate statistical approaches. Both of the two approaches were composed of pre-selection step (more sensitive, but less stringent) and refinement step (less sensitive, but more stringent). The pre-selection steps were used to find proteins that show a trend in differential expression between two experimental groups and therefore reduced numbers of multiple testing occurring in the refinement steps. The refinement steps aided to discover the strongest putative markers in the discovery study. The first approach consisted of ME-ANOVA pre-selection and *t*-test refinement (Step 2.1–2.3), which enabled finding proteins that were expressed in most of experimental samples but had significantly different abundance levels between two experimental groups. Therefore we defined this difference as “abundance” difference. The second approach combined Fisher’s exact test for pre-selection and *t*-test refinement (Step 2.4). It aimed to identify proteins preferentially expressed in one of the experimental groups but which were not necessarily detected in majority of the experimental samples, which was defined as “presence-absence” difference. However, low abundant proteins are often not reproducibly detected through the entire MS dataset due to undersampling issue of shotgun proteomics, even though these proteins are indeed present in all the samples. Therefore, some stably expressed low abundant species can be mistaken for putative markers only when presence and absence are taken into account instead of actual abundance of these proteins. To avoid high FDR, only the proteins that were also differentially expressed at the level of imputed protein abundances were regarded as putative candidates.
  2.1. ME-ANOVA test was performed on filtered peptides from Step 1.3, according to a previously described method [12, 13, 18]. This model takes into account four types of bias that may be introduced during the experimental procedure, known as experimental, group, peptide, and random error, and tries to calibrate these biases to achieve maximal separation between different experimental groups. In this model, higher abundant peptides assigned to certain protein weigh more than their lower abundant counterparts in estimating protein abundance. In our study, maximum 10 most abundant peptides per protein were used to test significance of their assigned proteins using a robust linear regression model in ME-ANOVA. However, it is difficult to estimate different biological and technical variations between the clinical samples since those samples were not collected under experimental conditions, especially no technical replicate was used for nLC-MS/MS profiling. Therefore, this model is only suitable for pre-selection of putative markers, and an additional step of consolidation is required to find truly differentially expressed proteins between different experimental groups;
  2.2. Type I error (false positive hits) introduced during multiple hypothesis testing was corrected for using Benjamini-Hochberg *p*-value adjustment [19]. In this way, differentially expressed proteins were found using a corrected *p*-value cutoff of 0.05;
  2.3. Next to the ME-ANOVA test, an additional *t*-test was performed on pre-selected putative markers identified by ME-ANOVA using their protein abundances to further refine the putative protein candidates (*p* < 0.05, permutation-based FDR = 0.05);
  2.4. In ME-ANOVA test and subsequently *t*-test, proteins present in only one of the experimental groups led to invalid test and therefore could not be captured. Therefore, a Fisher’s exact test was performed on MS/MS counts of all identified proteins except those recognized as reversed sequence (*p* < 0.05). In this way, proteins present in (mainly) one of the experimental groups could be discovered. Furthermore, data imputation was performed on the abundance of these differentially expressed proteins to replace missing values by normal distribution, and a *t*-test was performed on the imputed abundances to confirm differentially expressed patterns of these proteins (*p* < 0.05, permutation-based FDR = 0.05);
  2.5. Hierarchical clustering was performed on the abundance of differentially expressed proteins. For hierarchical clustering analysis, protein expression data were first centered based on their median abundances, followed by clustering both samples and proteins using Euclidean distance and complete linkage.
  Note: The filtering steps were performed in Microsoft Excel 2010. DanteR (version 1.0.1.1) and Perseus (version 1.2.0.17) were used to perform different types of statistical analysis including Log_2_ transformation, correlation plot, statistical tests, imputation, *p*-value adjustment, and volcano plot, while hierarchical clustering was executed using Cluster 3.0 and visualized in TreeView (version 1.1.5r2-win).

## Results and discussion

**Figure 1:**
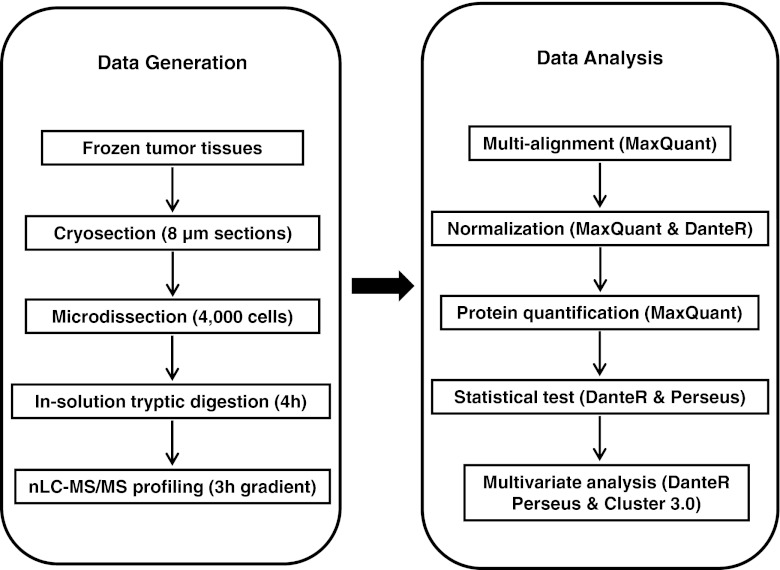
Flowchart summarizes the principle of label-free tissue proteomics pipeline. This technical platform is divided into two stages. The first stage generates nLC-MS/MS raw data from tumor tissues, and the second part proposes a general data processing procedure used in MS-based label-free proteomics biomarker discovery study

In this study, we describe a robust tissue proteomics pipeline for biomarker discovery, which enables identification of ER associated proteins in human breast cancer. The entire pipeline is divided into two different stages. The first stage consists of all procedures that generate raw nLC-MS/MS profiling data, while the latter part includes both upstream (multiple peak alignment, peptide and protein identification, and quantification) and downstream (statistical analysis) data handling steps. A flow chart summarizes the basic structure of this pipeline (Fig. 1).

To evaluate reproducibility of our tissue proteomics pipeline, we first inspected the number of identified peptides and protein groups of different samples. On average, 10,792 ± 275 (*x* ± *s*), 10,539 ± 742 and 10,374 ± 491 peptides were identified corresponding to 1,869 ± 40, 1,776 ± 98 and 1,869 ± 60 protein groups in 12 WTL-CTRLs, 5 LCM-CTRLs and 9 experimental samples, respectively, and identifications of razor and unique peptides in three types of samples were also roughly equivalent (Table 1). Furthermore, as expected, LCM-CTRLs had larger coefficient of variations (CVs) (7.0, 7.3, and 7.4 %) than WTL-CTRLs (2.6, 2.6, and 2.6 %) on peptide, razor peptide and unique peptide identifications, respectively (Table 1). Thus, our tissue proteomics pipeline was able to consistently identify similar numbers of peptides and proteins in the same type of tissue materials. Peptide identifications and their abundances in all the samples are listed in Supplementary table 1 and 2, and protein identifications and their abundances in all samples are listed in Supplementary table 3 and 4.

**Table 1:** Average numbers of identified peptides and protein groups

Category	WTL-CTRL samples	LCM-CTRL samples	Experimental samples
Total peptides	10,792 ± 275^a^ (2.6 %)^b^	10,539 ± 742 (7.0 %)	10,374 ± 491 (4.7 %)
Razor peptides	488 ± 10 (2.0 %)	534 ± 29 (5.4 %)	483 ± 17 (3.5 %)
Unique peptides	9,664 ± 254 (2.6 %)	9,263 ± 684 (7.4 %)	9,217 ± 472 (5.1 %)
Protein groups	1,869 ± 40 (2.1 %)	1,776 ± 98 (5.5 %)	1,869 ± 60 (3.2 %)

^a^Mean ± Standard deviation (*x* ± *s*);

^b^Percentages in brackets represent coefficient of variations of numbers of peptide and protein identification.

**Figure 2:**
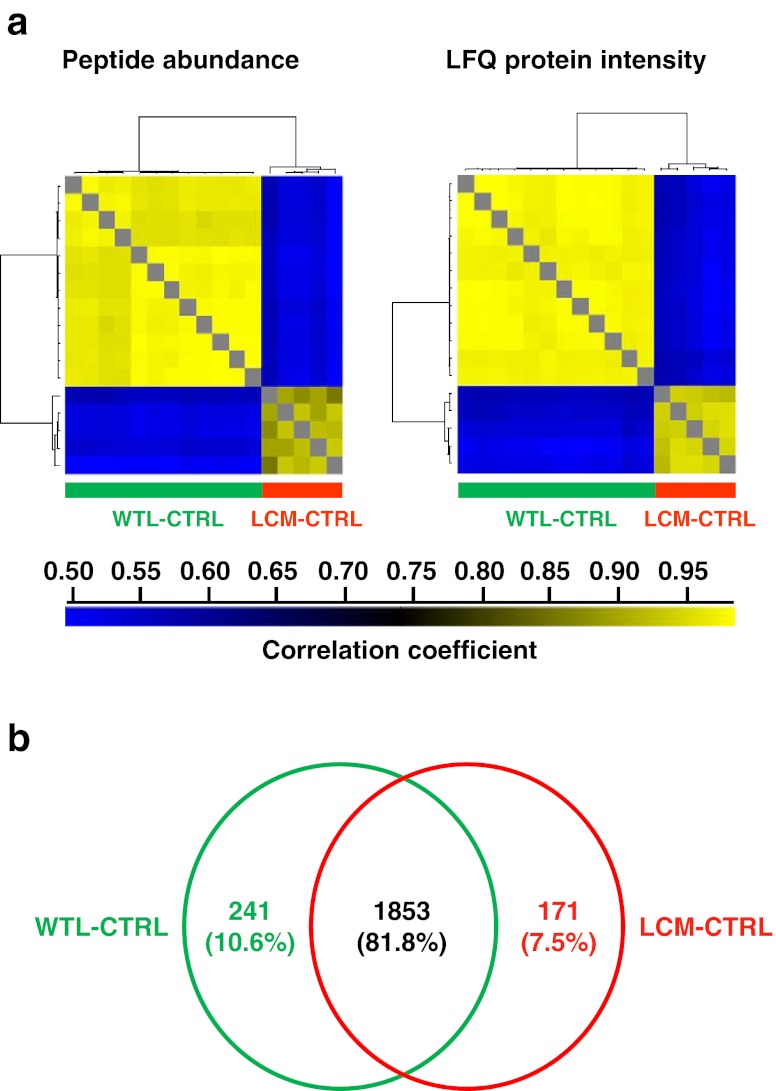
Application of label-free tissue proteomics pipeline to control and experimental breast cancer samples. **a** Pearson correlation of peptide and protein abundance between WTL-CTRLs and LCM-CTRLs; **b** A Venn diagram reveals shared and unique identified protein groups in WTL-CTRLs (*green circle*) and LCM-CTRLs (*red circle*)

Reproducibility of abundance data was first investigated through Pearson correlation of all LCM-CTRLs and WTL-CTRLs using their peptide abundances. On average, correlation between LCM-CTRLs was 0.91 ± 0.02 (*x* ± *s*), and a slightly higher correlation was observed between WTL-CTRLs (0.97 ± 0.01) (Fig. 2a, left panel). This indicates a good reproducibility of our sample preparation protocol and nLC-MS/MS analyses. Correlation of peptide abundances between different experimental samples was lower (0.72 ± 0.04) (data not shown), which can be explained by both technical variation (e.g.: different tissue quality and different morphology) and, more importantly, biological variation (e.g.: inter- and intra-tumor heterogeneity). Next, we inspected reliability of estimated protein abundance. An average higher correlation was observed between all LCM-CTRLs (0.94 ± 0.01) and WTL-CTRLs (0.98 ± 0.01) using LFQ protein abundances (Fig. 2a, right panel) compared to correlation of peptide abundances. Moreover, we also observed a good Pearson correlation between protein abundance and MS/MS counts of the same sample in both LCM-CTRL (0.84 ± 0.03) and WTL-CTRL (0.81 ± 0.02) (data not shown). Thus, the LFQ algorithm properly computed and normalized protein abundance.

Quality of our data generation workflow was further inspected through reproducibility of peptide identifications in WTL-CTRLs, LCM-CTRLs or experimental samples. Peptide identifications in ≤33 %, 34–66 %, and ≥67 % and of all samples were considered to be of low, medium or high reproducibility, respectively. In WTL-CTRLs, LCM-CTRLs, and experimental samples 80 %, 73 %, and 59 % of peptides were identified with high reproducibility, whereas 9 %, 14 %, and 23 % of peptides were identified with medium reproducibility, and 11 %, 13 %, and 18 % with low reproducibility, respectively (Supplementary Fig. 3a, upper panel). At the protein level, 85 %, 80 %, and 73 % of identifications was observed in more that 66 % of the samples (Supplementary Fig. 3a, lower panel). Furthermore, we observed that average CVs of the peptide abundances of WTL-CTRLs, LCM-CTRLs and experimental samples were 32.5 % ± 12.5 %, 64.1 % ± 24.8 % and 64.3 % ± 32.8 %, respectively (Supplementary Fig. 3b). Surprisingly, there was no significant difference in CVs of peptide abundances between LCM-CTRLs and experimental samples (*P* > 0.05), whereas there was a significant difference at the LFQ protein level (33.3 % ± 25.3 % and 81.3 ± 51.5 %, respectively, *P* < 0.000) (Supplementary Fig. 3b). In addition, CVs of WTL-CTRLs and LCM-CTRLs were overall lower in LFQ protein data (21.1 % ± 19.9 % and 33.3 % ± 25.3 %, respectively) than peptide data (32.5 % ± 12.5 % and 64.1 % ± 24.8 %, respectively), while CVs of experimental samples became larger after normalizing peptide abundance into protein abundance (64.3 % ± 32.8 % and 81.3 ± 51.5 %) (Supplementary Fig. 3b). These data indicate that the raw peptide abundance was properly normalized before further data mining, and further supports that LFQ algorithm properly normalized protein abundance. In conclusion, these observations suggest that reproducible data were generated using our tissue proteomics platform, and that upstream data analysis produced high quality data for further statistical analyses.

Next, WTL-CTRLs and LCM-CTRLs were compared to investigate the overlap in identified proteins. In total we identified 2,265 protein groups in 12 WTL-CTRLs and 5 LCM-CTRLs, of which 1,853 (81.8 %) were identified in both sample types (Fig. 2b). Only 241 (10.6 %) and 171 (7.5 %) protein groups were exclusively identified in either WTL-CTRLs or LCM-CTRLs, respectively (Fig. 2b). Unique proteins that were typically identified in WTL included most of major histocompatibility Class II molecules. These antigens are exclusively located on immune cells such as antigen-presenting cells and lymphocytes, which are only rarely microdissected along with tumor cells and thus not often identified in LCM samples. Furthermore, extracellular matrix proteins such as some collagens present in the stromal compartment were identified in WTL samples. Some of these proteins are highly abundant and may have caused undersampling during the MS profiling, meaning that their lower abundant co-elutes in WTL samples escaped MS/MS fragmentation and remained unidentified, explaining 7.5 % unique protein identification in LCM-CTRLs samples.

**Figure 3:**
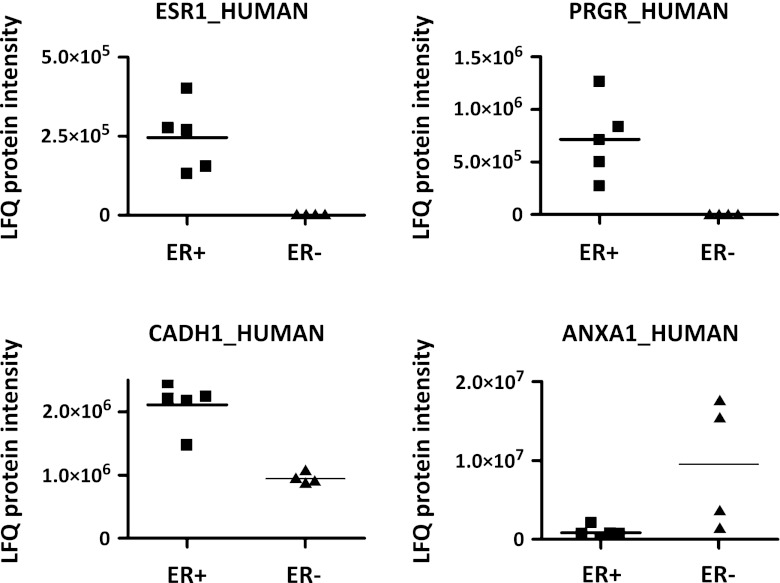
Four breast cancer related proteins and their expression in ER+ and ER− breast cancer samples

As a proof-of-principle, we applied the tissue proteomics and data handling pipeline to an experimental set of 5 ER+ and 4 ER− breast cancer tissues with the aim of identifying some known ER associated protein markers, in order to show that this platform can be used for large scale of biomarker discovery study. Protein abundance of ER (Entry name: ESR1_HUMAN), and downstream regulated proteins PR (Entry name: PRGR_HUMAN), Cadherin-1 (Entry name: CDH1_HUMAN) and Annexin A1 (Entry name: ANXA1_HUMAN), were investigated. As expected, ER and PR were completely absent in all 4 ER− samples (Fig. 3, upper panel), in concordance with in-house available enzyme-linked immunosorbent assay data from the same samples. Also, Cadherin-1 and Annexin A1 were elevated in ER+ and ER− samples (Fig. 3, lower panel), respectively. Cadherin-1, also known as E-cadherin, is well-known to mediate cell–cell adhesion, is important in breast cancer suppression, and is frequently down-regulated in ER− breast cancer cells [20]. Several clinical studies also showed that loss and aberrant expression of Cadherin-1 more frequently occurs in ER− breast cancer cases [21, 22], especially of the triple negative phenotype [23]. Also, Annexin A1 expression has been associated with breast cancer cell lines of the basal subtype [24], which are all ER−. In conclusion, these data indicate that the LFQ algorithm from MaxQuant was able to correctly determine relative protein abundance between different groups of breast cancer samples.

**Figure 4:**
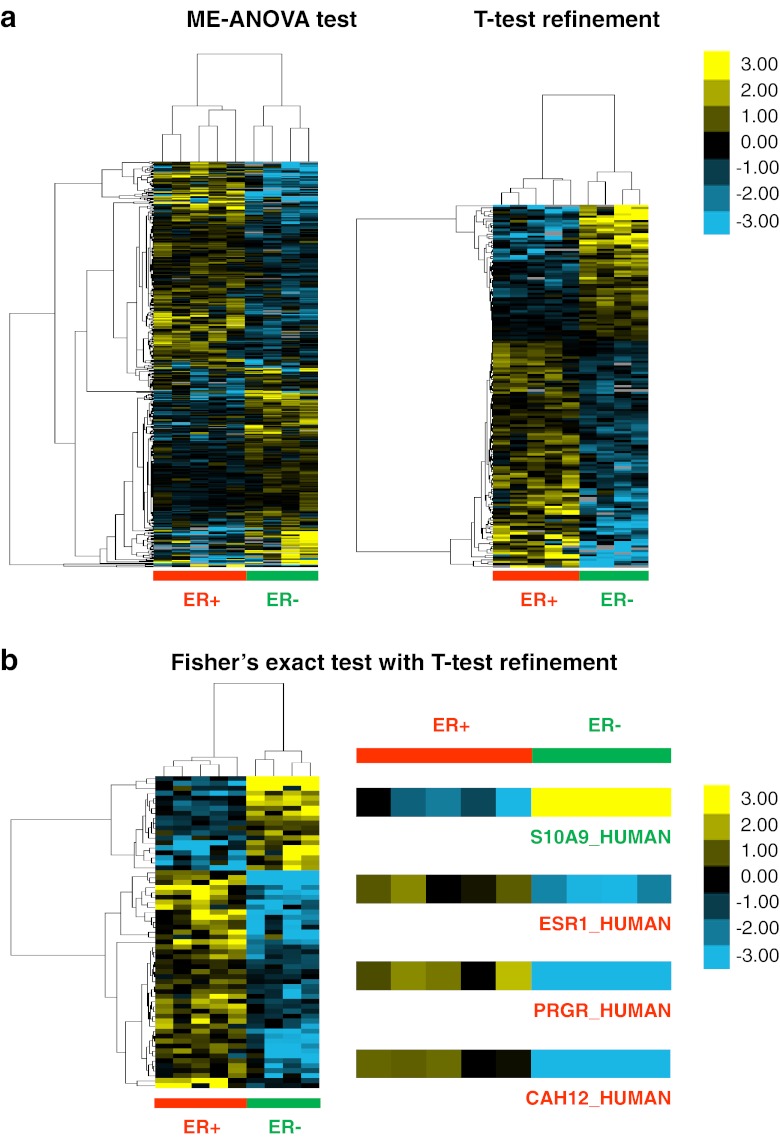
Differentially expressed proteins were discovered by different statistical analyses. **a** Hierarchical clustering separates ER+ and ER− samples using 435 (left panel) and 165 (right panel) differentially expressed proteins found by ME-ANOVA and refined using *t*-test; **b** Hierarchical clustering of 63 differentially expressed proteins between ER+ and ER− samples which were discovered using Fisher’s exact test with *t*-test refinement, as well as expression of 4 differentially expressed proteins out of these 63 proteins

In order to reveal differentially expressed proteins between ER+ and ER− breast tumors, two different comparative proteome analyses were performed: (1) revealing quantitative differences (ME-ANOVA with *t*-test refinement); and (2) revealing proteins more frequently expressed in one of the experimental arms compared to the other (Fisher’s exact test with *t*-test refinement). Using ME-ANOVA test, a total of 435 differentially expressed proteins were found between ER+ and ER− breast cancer samples (*p* < 0.05). ER+ and ER− samples were well separated into two clusters using these 435 protein markers (Fig. 4a, left panel). However, the protein dendrogram (vertical axis) did not nicely fall into two clusters, indicating some low discriminatory proteins were also included in this clustering. To further refine the analysis to find the strongest markers, a *t*-test was used on Log_2_ intensity values of 435 proteins to confirm differences between ER+ and ER− samples. With this more stringent refinement, 165 proteins were confirmed as differentially expressed proteins between ER+ and ER− samples (*p* < 0.05, permutation-based FDR = 0.05) (Supplementary table 5). Those 165 proteins formed a more solid protein dendrogram with two major arms (Fig. 4a, right panel). These findings suggest that ME-ANOVA with *t*-test refinement can reliably identify differentially expressed proteins between ER+ and ER− breast cancer samples.

Additionally to reveal proteins more frequently present in one of the experimental arms, MS/MS counts of all identified proteins were subsequently analyzed by a Fisher’s exact test in order to identify protein markers that are mainly present in one of the experimental groups. In total, 102 differentially expressed proteins were discovered using this approach. We further consolidated 63 proteins by performing a *t*-test on imputed Log_2_ intensity of these 102 proteins using a *p*-value cutoff of 0.05 and permutation-based FDR of 0.05 (Supplementary table 6). ER+ and ER− samples could also be correctly classified using these 63 proteins (Fig. 4b). As expected, we found that ER and PR were enriched in ER+ breast cancer samples. Also, carbonic anhydrase 12 (Entry name: CAH12_HUMAN) and Protein S100-A9 (Entry name: S10A9_HUMAN) were augmented in ER+ and ER− breast cancer samples, respectively (Fig. 4b). Carbonic anhydrase 12 was documented as an ER regulated protein in breast cancer, and expression level of this protein is highly positively correlated to expression level of ER [25, 26]. Protein S100-A9 has been associated with basal breast cancer which are typically ER− [27]. Moreover, 31 out of 63 differentially expressed proteins were also identified by ME-ANOVA test with *t*-test refinement with the same direction of regulation in ER+ and ER− samples (Supplementary table 7), which demonstrates validity of two types of statistical approaches, but both methods also provided complementary advantages in discovering putative markers. Therefore, ER, PR and some other ER associated proteins were only identified using combination of Fisher’s exact test and imputation-based *t*-test refinement, indicating this approach is of added value to the quantitative statistical analysis.

In summary, we described a highly reproducible and robust label-free tissue proteomics pipeline for MS-based biomarker discovery. This platform produced high-quality MS data from as little as ∼4,000 LCM breast tumor epithelial cells and reliably quantified protein abundance from observed peptide abundance. More importantly, it allowed identification of a large number of differentially expressed proteins between different experimental groups under investigation with relatively low FDR. Some of these differentially expressed proteins were previously described in literature as markers for ER+ or ER− breast cancer. Therefore, we conclude that this label-free tissue proteomics pipeline is suitable for clinical biomarker discovery.

## Electronic supplementary material

Below is the link to the electronic supplementary material.

Supplementary Fig. 1 Detailed sampling plan and experimental design of the current study. (PDF 67 kb)

Supplementary Fig. 2 Comprehensive statistical strategy applied in this label-free tissue proteomics pipeline. (PDF 54.5 kb)

Supplementary Fig. 3 Reproducibility of label-free tissue proteomics pipeline. (a) Pie charts represent percentage of peptide and protein identifications in WTL-CTRLs, LCM-CTRLs or experimental samples with low (blue area), medium (red area) and high (green area) reproducibility. The reproducibility was defined based on how many times a certain peptide or protein was observed between different individuals in each type of samples. Peptides or proteins observed in ≤33 %, 34–66 %, and ≥67 % of the measurements in each type of samples were defined as low, medium and high reproducibility, respectively; (b) A box plot shows distribution of CVs of non-Log_2_ transformed peptide and LFQ protein abundances. Only peptides and proteins identified in multiple WTL-CTRLs, LCM-CTRLs or experimental samples were taken into account for calculation of CVs. Red and pink asterisks represent outliers of CVs. P values: ***: < 0.000, N.S: > 0.05. (PDF 92.9 kb)

Supplementary table 1 Peptide intensity of LCM-CTRL and WTL-CTRL samples (XLSX 3033 kb)

Supplementary table 2 Peptide intensity of experimental samples (XLSX 2309 kb)

Supplementary table 3 Protein intensity of LCM-CTRL and WTL-CTRL samples (XLSX 1085 kb)

Supplementary table 4 Protein intensity of experimental samples (XLSX 776 kb)

Supplementary table 5 165 differentially expressed proteins identified by ME-ANOVA test and t-test refinement (XLSX 32 kb)

Supplementary table 6 63 differentially expressed proteins identified by Fisher’s exact test and t-test refinement (XLSX 23 kb)

Supplementary table 7 31 differentially expressed proteins identified by both ME-ANOVA with t-test refinement and Fisher’s exact test with t-test refinement (XLSX 19 kb)

## Abbreviations

AGC: Automatic gain control
CVs: Coefficient of variations
ER+/−: Estrogen receptor α positive/negative
FDR: False discovery rate
LCM: Laser capture microdissection/microdissected
LCM-CTRLs: Laser capture microdissected control samples
LFQ: Label-free quantitation
LTQ-Orbitrap-XL: Linear ion trap/Orbitrap mass spectrometer
ME-ANOVA: Mixed-effect analysis of variance model
MS: Mass spectrometry
m/z: Mass-to-charge ratio
nLC-MS/MS: Nanoscale liquid chromatography coupled to tandem mass spectrometry
PEN: Polyethylene naphthalate
PR: Progesterone receptor
WTL: Whole tissue lysate
WTL-CTRLs: Whole tissue lysate control samples
