# Supplementary figures and images for: Proteomics Pipeline for Biomarker Discovery of Laser Capture Microdissected Breast Cancer Tissue
Source: J Mammary Gland Biol Neoplasia. 2012 May 30;17(2):155–64. doi: 10.1007/s10911-012-9252-6 (PMC3428526; doi:10.1007/s10911-012-9252-6)

**Supplementary Fig. 1**

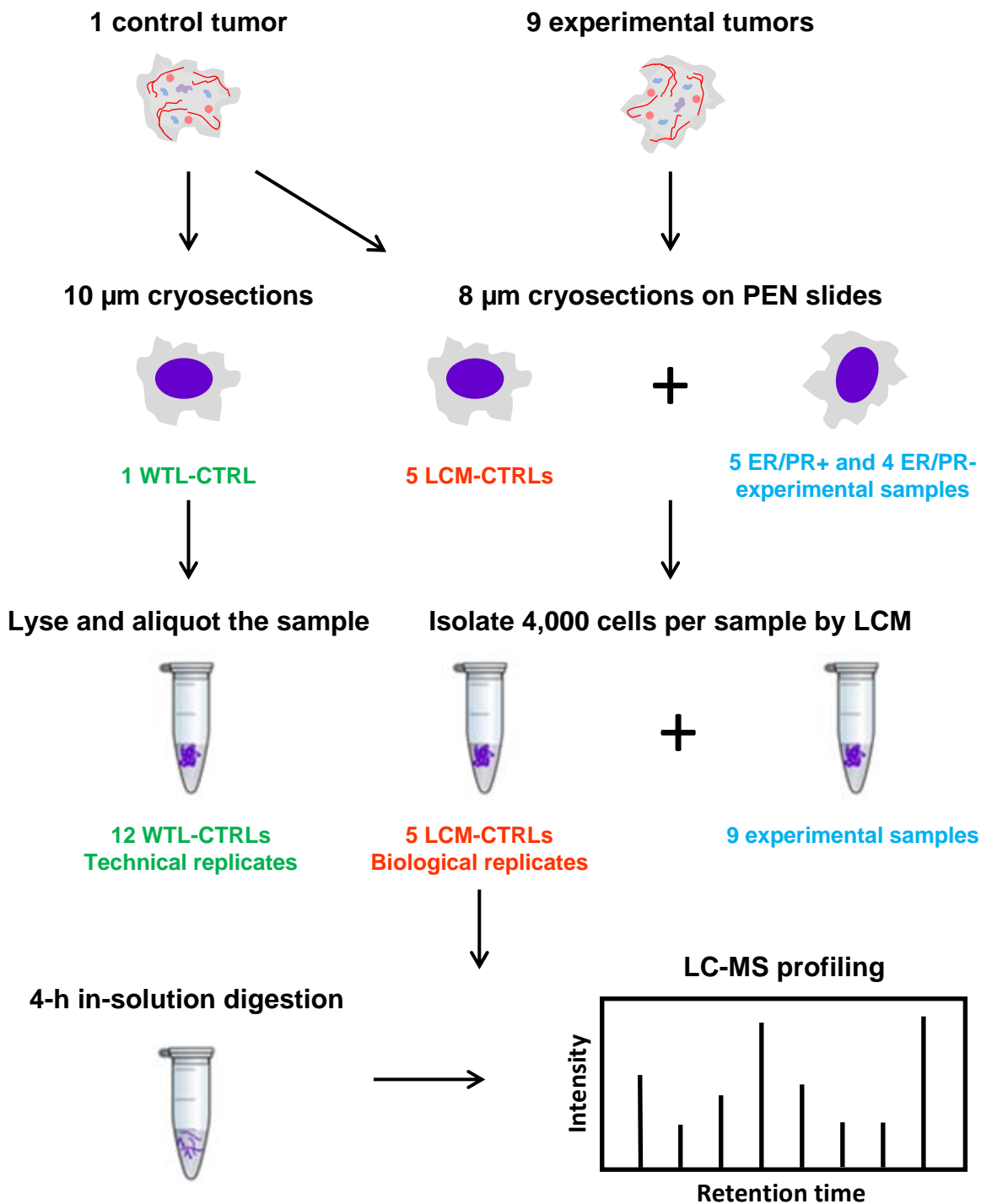

Supplement: Supplementary file 1 — Detailed sampling plan and experimental design of the current study. (PDF 67 kb) [file 10911_2012_9252_MOESM1_ESM.pdf]

Supplementary Fig. 2

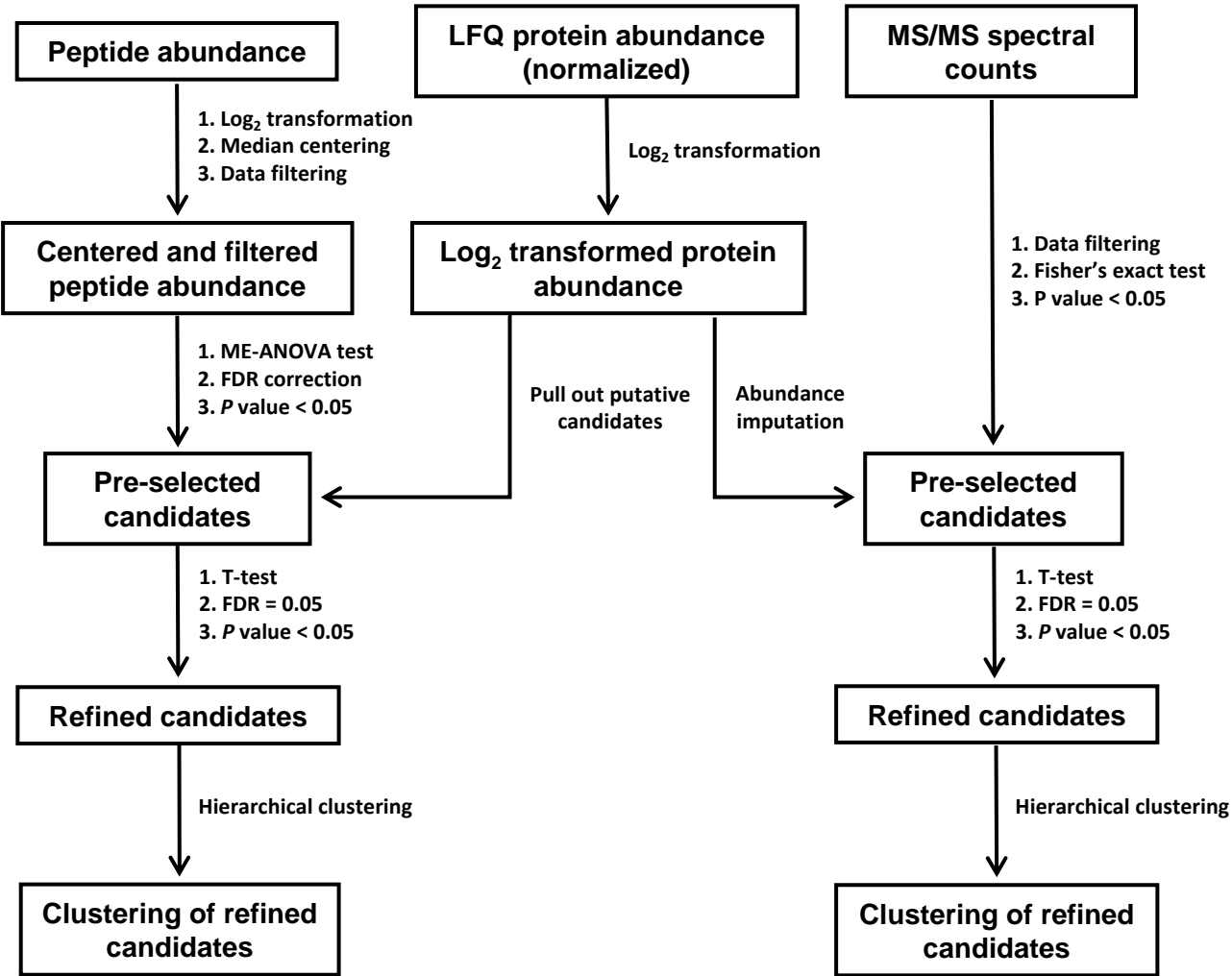

Supplement: Supplementary file 2 — Comprehensive statistical strategy applied in this label-free tissue proteomics pipeline. (PDF 54.5 kb) [file 10911_2012_9252_MOESM2_ESM.pdf]

Supplementary Fig. 3

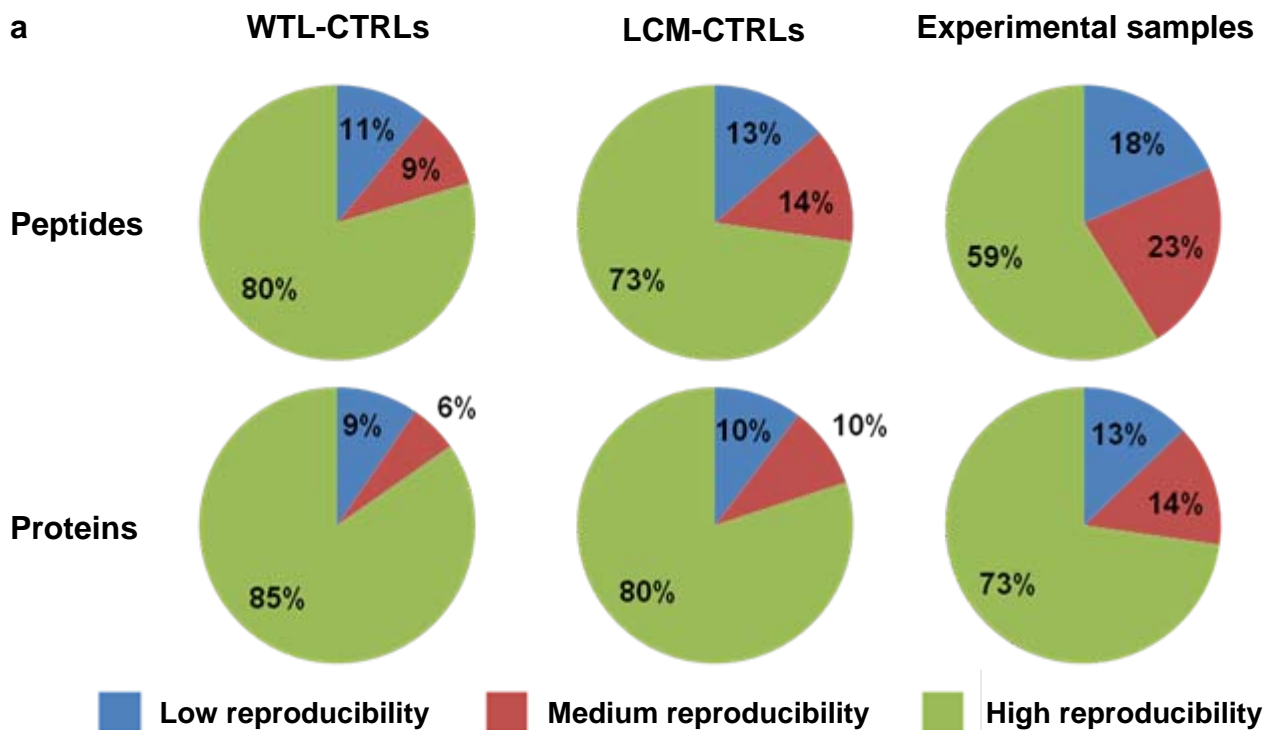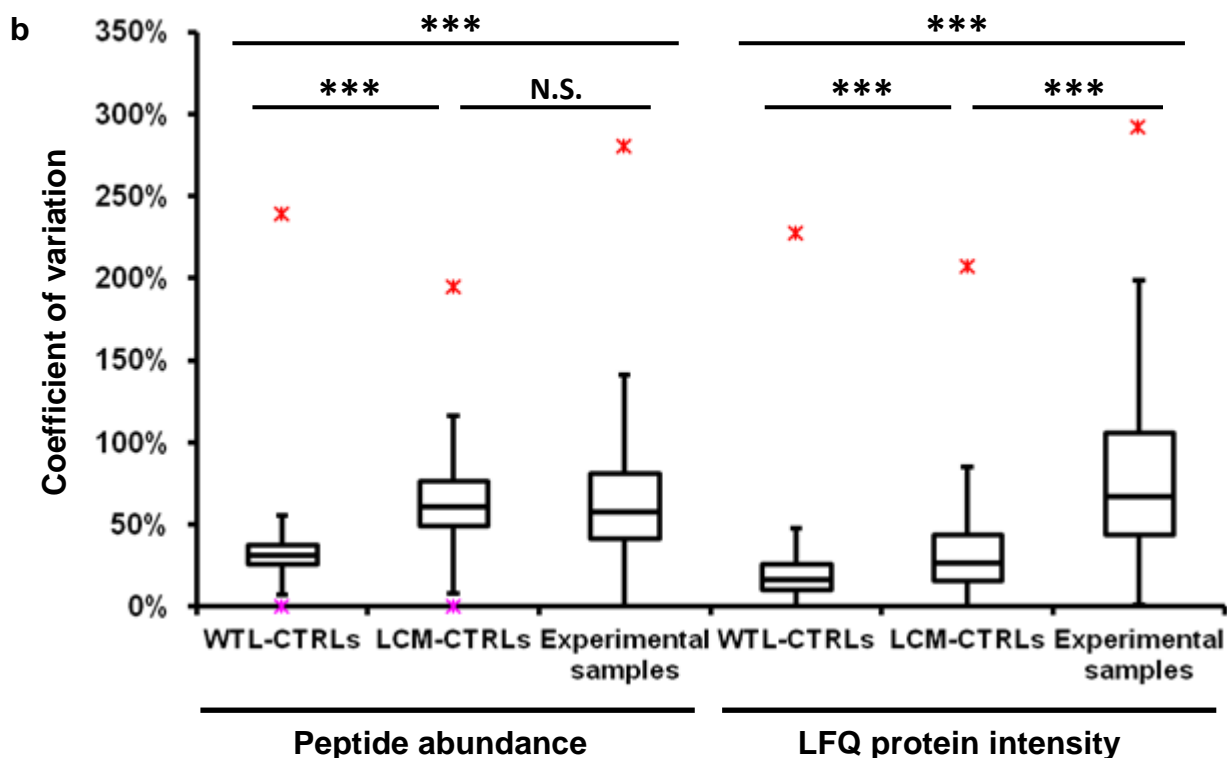

Supplement: Supplementary file 3 — Reproducibility of label-free tissue proteomics pipeline. (a) Pie charts represent percentage of peptide and protein identifications in WTL-CTRLs, LCM-CTRLs or experimental samples with low (blue area), medium (red area) and high (green area) reproducibility. The reproducibility was defined based on how many times a certain peptide or protein was observed between different individuals in each type of samples. Peptides or proteins observed in ≤33 %, 34–66 %, and ≥67 % of the measurements in each type of samples were defined as low, medium and high reproducibility, respectively; (b) A box plot shows distribution of CVs of non-Log2 transformed peptide and LFQ protein abundances. Only peptides and proteins identified in multiple WTL-CTRLs, LCM-CTRLs or experimental samples were taken into account for calculation of CVs. Red and pink asterisks represent outliers of CVs. P values: ***: < 0.000, N.S: > 0.05. (PDF 92.9 kb) [file 10911_2012_9252_MOESM3_ESM.pdf]
